# Lack of evidence of HPV etiology of prostate cancer following radical surgery and higher frequency of the Arg/Pro genotype in turkish men with prostate cancer

**DOI:** 10.1590/S1677-5538.IBJU.2015.0429

**Published:** 2017

**Authors:** Merve Aydin, Aliseydi Bozkurt, Aytekin Cikman, Baris Gulhan, Mehmet Karabakan, Aysun Gokce, Murat Alper, Murat Kara

**Affiliations:** 1 Department of Medical Microbiology, Faculty of Medicine, Erzincan University, Erzincan, Turkey;; 2 Department of Urology, Erzincan University, Mengucek Gazi Training and Research Hospital, Erzincan, Turkey;; 3 Department of Pathology, Dıskapı Training and Research Hospital, Ankara, Turkey

**Keywords:** Papillomaviridae, Prostatic Neoplasms, Tumor Suppressor Protein p53

## Abstract

**Objectives:**

The aim of this study was to assess the possible role of HPV in the development of prostate cancer (PCa) and investigate the distribution of the p53 codon 72 polymorphism in PCa in a Turkish population.

**Materials and methods:**

A total of 96 tissues, which had been obtained using a radical surgery method, formalin-fixed and parafin-embedded, were used in this study. The study group consisted of 60 PCa tissues (open radical prostatectomy) and the control group contained 36 benign prostatic hyperplasia tissues (BPH) (transvesical open prostatectomy). The presence of HPV and the p53 codon 72 polymorphism was investigated in both groups using real-time PCR and pyrosequencing.

**Results:**

The results of the real-time PCR showed no HPV DNA in any of the 36 BPH tissue samples. HPV-DNA was positive in only 1 of the 60 PCa samples (1.7%). The HPV type of this sample was identified as HPV-57. The distribution of the three genotypes, Arg/Arg, Arg/Pro and Pro/Pro was found to be 45.6, 45.6, and 8.8% in the PCa group and 57.1%, 34.3% and 8.6% in the control group, respectively. Compared with the control group, patients with PCa had a higher frequency of the Arg/Pro genotype and Proline allele (odds ratio (OR)=1.67, 95% confidence interval (CI)=0.68-4.09, p=0.044; OR=1.13, 95% CI=0.76-1.68, p=0.021, respectively).

**Conclusions:**

The results of the study do not support the hyphothesis that prostate cancer is associated with HPV infection but indicated that Proline allele can be a risk factor in the development of PCa in the Turkish population.

## INTRODUCTION

Prostate cancer (PCa) is the most common non cutaneous cancer and the second leading cause of male cancer-related death in the Western countries (1). Risk factors in the development of PCa have been clearly identified as ethnic origin, age, environment and genetic factors (2). In addition, in recent years, it has been reported that sexually transmitted diseases increase the risk of PCa, and genetic and epigenetic changes as well as cell transformation can cause inflammation in the prostate (3, 4).

The prostate can be a target for human papillomavirus (HPV) infections due to its anatomical location (4). Human papillomaviruses (HPVs) are a small and non-enveloped viruses that contain double stranded DNA genomes of approximately 8.000 base-pairs (bp). Of the nearly 200 HPV that have so far been identified, one-third can cause infections in the genital system (5, 6). While low-risk HPV types cause benign lesions, high-risk HPV types such as 16, 18, 26, 31, 33, 35, 39, 45, 51, 52, 53, 59, 66, 68, 72 and 81 have carcinogenic potential (6). High-risk HPV types have also been reported particularly in cervical cancer as well as vulvar, vaginal, penile and anal cancers (7). E6 and E7 viral proteins play a significant role in the carcinogenic process where HPV acts as an intermediary. The E6 protein binds to the p53 tumor supressor gene and results in its degradation. The E7 gene bind to the retinoblastoma gene protein of tumor supressors and inactivates it (6).

Many studies have been conducted to explore the association between HPV infection and PCa; however, the possible role of HPV infection in the pathogenesis of PCa remains controversial (8-14). Many researchers have reported that HPV infection has a positive correlation with PCa and increases the risk of PCa (8-10, 13). However, there are also researchers who have suggested that there is no association between HPV infection and the pathogenesis of the PCa (11, 12, 14).

The most commonly mutated p53 tumor suppressor gene is located on chromosome 17p13 and encodes the tumor supressor protein called the “guardian of the genome”. This protein has an important role in many cellular process such as cell cycle arrest, DNA repair and apoptosis (15, 16).

The p53 gene has several single-nucleotide polymorphisms. The most common is the codon 72 polymorphism located on the exon 4 (Arg72Pro, rs1042522 G>C). In the 72nd codon of the p53 gene, only one nucleotide alternates the amino acid from Arginine (Arg) (CGC) to Proline (Pro) (CCC). This alternation of the amino acid affects the biochemical and functional characteristics of the p53 protein. The Pro variant strongly activates transcription; however, the Arg variant induces the apoptosis (16, 17).

Despite the abundance of studies on the association between PCa and the p53 codon 72 polymorphism, the results are contradictory (10, 18-22). In some studies, the Arg genotype has been reported to increase the development of PCa (22) while only one study has shown that the Pro genotype reduces the risk of PCa (18).

The aim of the current study was to explore the presence of HPV and the distribution of the p53 codon 72 polymorphism in surgical specimens of localized prostate cancer in a Turkish population using real-time PCR and pyrosequencing methods.

## MATERIALS AND METHODS

### Sample of the Study

Formalin-fixed paraffin-embedded (FFPE) tissue samples were selected from July 2011 to June 2014 archive collection of the Pathology Laboratory of the Dıskapi Training and Research Hospital, Ankara. To eliminate the possibility of urethral and anal HPV contamination, only samples that had been obtained through radical surgery method were included in the study. The study group consisted of 60 prostate adenocarcinoma tissues (open radical prostatectomy) and the control group was composed of 36 benign prostatic hyperplasia (BPH) tissues (transvesical open prostatectomy). All the study and control cases were of Turkish ethnic origin. None of the patients had received neoadjuvant radiotheraphy or chemotheraphy. The study was approved by the Ethics Committee of Erzincan University.

### Tumor Samples

Tissue sections of 3µm wide were stained using hematoxylin eosin and the slides were evaluated by two expert pathologists in terms of the presence of cancer and confirmation of the first histological diagnosis. The histological sections were than manually dissected to determine the area of the tumor for DNA extraction. From each block, 5-10µm sections were obtained for DNA extraction. Samples displaying the characteristics of adenocarcinoma cell infiltration or hyperplasia were selected. These steps were repeated as required until 90% of neoplastic cells were visible.

Tumors were graded according to the Gleason scoring system, and staging was performed according to the 2009 TNM classification (23). Finally, the tumors were assessed using the new grading system for prostate cancer proposed by the International Society of Urological Pathology (ISUP) in 2014 (24). Table-1 presents the demographic and pathologic characteristics of the study samples.

**Table 1:** Clinico-pathological characteristics of the patients.

Variable	Samples	

	PCa (n=60)	BPH (n=36)
**Mean age (SD)**	63.04±6.67	67.94±8.04
**Surgery**		
Open Radical Prostatectomy	60 (100%)	0
Transvesical Open Prostatectomy	0	36 (100%)
**Gleason Score**		
Gleason ≤6	29 (48.3%)	-
Gleason 7	24 (40.0%)	-
Gleason ≥8	7 (11.7%)	-
**New Grading System**		
Group 1	29 (48.3%)	-
Group 2	14 (23.3%)	-
Group 3	10 (16.7%)	-
Group 4	3 (5.0%)	-
Group 5	4 (6.7%)	-
**Pathologic Stage**		
T1	0	-
T2	29 (48.3%)	-
T3	30 (50.0%)	-
T4	1 (1.7%)	-
**Regional lymph nodes**		
Absent	59 (98.3%)	-
Present	1 (1.7%)	-
**Distant metastasis**		
Absent	0	-
Present	0	-

**PCa** = prostate cancer; **BPH** = benign prostatic hyperplasia

## HPV DNA ISOLATION AND DETECTION

### DNA Isolation

Tissue sections of 5-10µm thickness were cut from each tissue block and placed in 2mL sterile tubes. For deparaffinization, the samples were incubated in 1mL xylene for 10 seconds, centrifuged at 12.000rpm for 2 minutes and the supernatant was removed. To remove the remaining xylene, the samples were washed with 1mL absolute ethanol. Following a 2-minute centrifuge at 12.000rpm, the pellets were air-dried for 10 minutes to remove the ethanol. The genomic DNA isolation from the FFPE tissue samples was performed using a QIAamp DNA FFPE Tissue Kit (Qiagen, Hilden, Germany) according to the manufacturer’s recommendations. DNA was eluted in 50µL of buffer ATE and the DNA concentration was measured using the NanoDrop ND-1000 instrument (ThermoScientific, Wilmington, DE, USA), and then stored at-20ºC until use.

### HPV Genotyping

The presence of HPV in the tissue samples was investigated using mixed primers based on broad-spectrum HPV-DNA amplification and targeting the variable region of HPV L1 ORF. A real-time PCR (EVA GreenTM chemistry) and an HPV sign® Q24 complete kit (Diatech Pharmacogenetics, Italy) able to identify HPV types in a broad spectrum in the Rotor Gene machine were utilized. To control inhibition, the primer set included with the kit for the detection of Human Beta-globin (β-globin) was used. The HPV sign® Q24 complete kit was used according to the manufacturer’s recommendations. Each experiment contained at least one negative amplification control (water), HPV sign® positive control and h-DNA control (β-globin control). The presence or absence of HPV DNA was determined using a melting curve analysis. Pyrosequencing was performed on samples that were found to be HPV positive in the melting curve analysis using four specific sequencing primers and the Pyromark Q24 pyrosequencing instrument (Qiagen, Switzerland). Genotype-specific sequencing primers that allowed for the synthesis of 30 base sequences were chosen. These sequences were compared with other sequences in the HPV library and identified using the HPV genotype IdentiFire^TM^ software version 1.0.5.0 (Biotage AB, Uppsala, Sweden).

## GENOTYPING OF THE TP53 GENE AT CODON 72

### DNA isolation and PCR

The genomic DNA isolation for p53 genotyping was performed as described in the previous section. Four samples were not genotyped due to low DNA quantities. The primers were designed using the pyrosequencing assay design software 2.0 (Qiagen, Hilden, Germany). The PCR amplification of a 155-bp fragment of the p53 gene exon 4 was performed using forward (5’-AGACCCAGGTCCAGATGAAGC) and reverse (5’-biotin CGTAGCTGCCCTGGTAGGTT) primers (Biomers, Germany) with the PyroMark PCR Kit (Qiagen, Hilden, Germany). PCR reactions were performed in a 25µL mix containing 20ng genomic DNA and 5mM of each primer using the following protocol: initial denaturation at 95ºC for 15 min., followed by 45 cycles of 95ºC for 30 sec, 62ºC for 30 sec and 72ºC for 30 sec, and a final extension for 10 min. at 72ºC.

### Pyrosequencing

**Figure 1:**
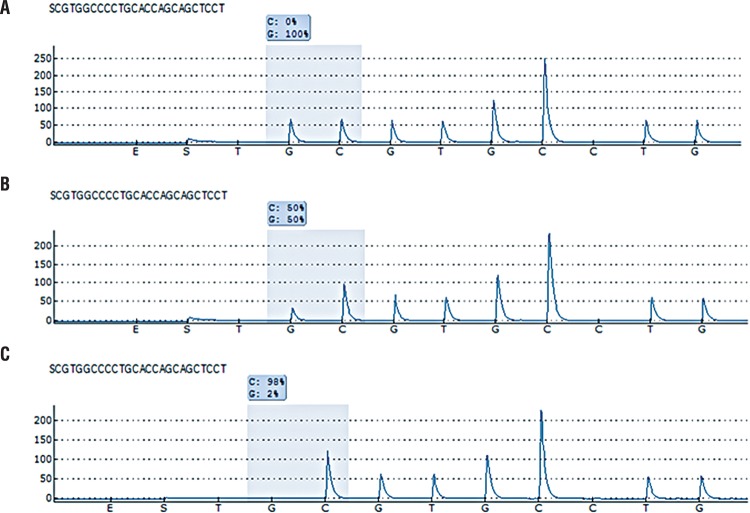
The raw pyrosequencing data for the allelic variants of p53 codon 72 polymorphism (A) arginine homozygote (B) arginine/proline heterozygote (C) proline homozygote.

Pyrosequencing reactions were performed using the PyroMark Gold Q24 reagents (Qiagen, Hilden, Germany) and the PyroMark Q24 instrument according to the manufacturer’s recommendations. For the pyrosequencing, single-stranded DNA templates were obtained using PyroMark Q24 Vacuum Prep Workstation (Qiagen, Hilden, Germany) according to the manufacturer’s recommendations. Briefly, a 10µL PCR product was immobilized in Streptavidin-coated Sepharose High Performance beads (GE Healthcare, Uppsala, Sweden), and processed to obtain single-stranded DNA. The template DNA was incubated at 80°C in a heat block for 2 min. with 25μL of 0.3μmol/L sequencing primer (5′-ATGCCAGAGGCTGCTCCCC) specific for codon 72. To assess the quality of genotyping and raw data, the PyroMark Q24 software (Qiagen, Hilden, Germany) was used (Figure-1).

### Statistical analysis

The data was analyzed using the SPSS 17.0 (Statistical Package for Social Sciences, SPSS Inc., Chicago, IL, USA). For the analysis of categorical values, the chi-square test (χ2 test) and Fisher’s exact test were used. p<0.05 was regarded as statistically significant. The chi-square (χ2) test was performed to observe the frequency of the genotypes. The expected values of the genotype frequencies were calculated using the Hardy Weinberg equilibrium (HWE). The odds ratio (OR) and its 95% confidence interval (CI) were determined to measure the correlation between p53 codon 72 Arg/Pro polymorphism in study and control groups.

## RESULTS

### Presence of HPV DNA in prostate tissues

A total of 96 male patients, 60 with PCa and 36 with BPH, were included in the study. 13.3% of patients with PCa and 44.4% of BPH patients were aged above 70. The mean age of total cohort was 63.04±6.67 years (range 48-75) and 67.94±8.04 years (range 53-89) in the PCa and BPH patients, respectively (p=0.089). Of the PCa patients, 29 (48.3%) were in stage T2, 30 (50%) in T3 and 1 (1.7%) was in T4. Furthermore, the Gleason score was found to be ≤6 in 29 patients (48.3%), 7 in 24 patients (40.0%), and ≥8 in the remaining 7 patients (11.7%). According to ISUP’s new grading system, 29 (48.3%) patients with prostate cancer were classified as Group 1, 14 (23.3%) as Group 2, 10 (16.7%) as Group 3, 3 (5.0%) as Group 4, and 4 (6.7%) as Group 5.

The presence of HPV DNA was investigated in 60 (62%) PCa tissues and 36 (38%) BPH tissues. To assess the quality of isolated DNA, the β-globin gene was amplified using the real-time PCR method using the HPV sign^®^ Q24 complete kit. In the Rotor gene Q analysis, all 96 samples (100%) were found to be positive in terms of human β-globin. No HPV DNA was detected using the real-time PCR (EVA Green^TM^ chemistry) with mixed primers targeting a hypervariable region of the HPV L1 ORF in any of the 36 BPH samples. One of the sixty (1.7%) PCa samples was found to be positive for HPV DNA using real-time PCR. Pyrosequencing analysis showed this sample had one type of HPV, namely HPV-57. The HPV-positive sample was classified as pT2cN0M0 and had a low Gleason score (final score 6).

### The p53 codon 72 Arg/Pro polymorphism

Due to the low quantity of the isolated DNA, p53 genotyping could not be performed on three PCa samples and one BPH sample. Table-2 presents the genotype frequency in the remaining study and control groups.

**Table 2:** Genotype and allele frequencies of the p53 codon 72 polymorphism in cancer patients and controls

p53 Arg72Pro	PCa (n=57) n (%)	BPH (n=35) n (%)	p value	OR (%95 CI)
**Genotypes**				
Arg/Arg	26 (45.6)	20 (57.1)	-	1.0 (reference)
Pro/Pro	5 (8.8)	3 (8.6)	0.337	1.28 (0.27-6.01)
Arg/Pro	26 (45.6)	12 (34.3)	0.044*	1.67 (0.68-4.09)
**Alleles**				
Arg	31 (72.1)	27 (77.1)	-	1.0 (reference)
Pro	12 (27.9)	8 (22.9)	0.021*	1.13 (0.76-1.68)

**p53 Arg72Pro**=p53 codon 72 polymorphism; **PCa**=prostate cancer; **BPH**=benign prostatic hyperplasia; **OR**=odds ratio; **CI**=confidence interval; **Arg**=Arginine; **Pro**=Proline;

* p<0.05, considered as statistically significant.

The genotypes identified in the p53 codon 72 polymorphism of 57 PCa samples were distributed as follows; Arg/Arg genotype in 26 samples (45.6%), Pro/Pro genotype in 5 (8.8%) and Arg/Pro genotype in 26 (45.6%). Of the 35 BPH samples, 20 (57.1%) had the Arg/Arg genotype, 3 (8.6%) had the Pro/Pro genotype, and 12 (34.3%) had the Arg/Pro genotype.

Comparing the study and control groups based on these results, the Arg/Pro genotype was more frequently seen in patients with PCa than those with BPH and the difference was statistically significant (OR=1.67, 95% CI=0.68-4.09, p=0.044). However, no statistically significant difference was found between PCa and control groups in terms of the frequency of the Arg/Arg and Pro/Pro genotypes (p=0.207; OR=1.28 95% CI=0.27-6.01, p=0.337, respectively).

The frequency of the Arg allele in study and control groups was 72.1% and 77.1%, respectively. Pro allele was observed in 27.9% of PCa patients and 22.9% of BPH cases. In this study, it was also found that the frequency of Pro allele was higher in the PCa patients, thus it can be postulated that Pro allele is associated with PCa (OR=1.13, 95% CI=0.76-1.68, p=0.021).

The p53 codon 72 polymorphism in patients with prostate cancer was investigated comparing the results from Gleason score, ISUP’s new grading system and pathologic staging (Table-3). The highest Gleason score was found to be ≤6 for the Arg/Pro and Arg/Arg genotypes while it was 7 for the Pro/Pro genotype. However, there was no statistically significant relationship between the p53 codon 72 polymorphism and the Gleason score (p=0.305). According to the results of the ISUP’s new grading system, most of the Arg/Pro and Arg/Arg genotypes were classified as Group 1 while Group 2 was predominant for the Pro/Pro genotype. However, no statistically significant correlation was found between the p53 codon 72 polymorphism and the new grading system (p=0.679). The most frequent pathologic stage was T3 for the Arg/Arg and Pro/Pro genotypes, and T2 for the Arg/Pro genotype. Similar to the results of other methods, the p53 codon 72 polymorphism did not have any significant correlation with pathologic stage (p=0.301).

**Table 3:** Comparison of the results on the p53 codon 72 polymorphism obtained from Gleason score, ISUP’s new grading system and pathologic stage in patients with prostate cancer

			p53 codon 72 polymorphism			Total	p

			Arg/Arg	Pro/Pro	Arg/Pro		
**Gleason score**	≤6	n	13	1	15	29	0.305
		%	50.0%	20.0%	57.7%	50.9%	
	7	n	9	4	8	21	
		%	34.6%	80.0%	30.8%	36.8%	
	≥8	n	4	0	3	7	
		%	15.4%	0%	11.5%	12.3%	
**New grading system**	Group 1	n	13	1	15	29	0.679
		%	50.0%	20.0%	57.7%	50.9%	
	Group 2	n	5	3	6	14	
		%	19.2%	60.0%	23.1%	24.6%	
	Group 3	n	4	1	2	7	
		%	15.4%	20.0%	7.7%	12.3%	
	Group 4	n	2	0	1	3	
		%	7.7%	0%	3.8%	5.3%	
	Group 5	n	2	0	2	4	
		%	7.7%	0%	7.7%	7.0%	
**Pathologic stage**	T2	n	12	1	15	28	0.301
		%	46.2%	20.0%	57.7%	49.1%	
	T3	n	14	4	10	28	
		%	53.8%	80.0%	38.5%	49.1%	
	T4	n	0	0	1	1	
		%	0%	0%	3.8%	1.8%	

Total		n	26	5	26	57	
		%	100.0%	100.0%	100.0%	100.0%	

**p53 Arg72Pro**=p53 codon 72 polymorphism; **Arg**=Arginine; **Pro**=Proline

## DISCUSSION

PCa is the most commonly diagnosed cancer in men. However, the etiology and molecular pathobiology of PCa is still not clear. The viral etiology of prostate carcinogenesis, which includes environmental, endogenous and genetic risk factors as well as HPV, is controversial (25).

HPV DNA was first detected in PCa and BPH tissues using PCR in 1990 in a study by McNicol and Dodd; however, the authors did not find a significant difference between the two groups (26). The controversial findings of this study resulted in several other researchers to become interested in this area and carry out further research.

From 1990 to December 2014, there are 40 papers in the literature (excluding case reports and reviews) reporting on the results of tissue-based studies investigating the association between HPV infection and PCa. Of these, thirty were case-control studies while the remaining 10 only analysed the PCa samples. The prevalence of HPV infection in PCa samples has been reported in range of %0 to 100% (27).

Only 4 of the 40 studies suggested a potential association between the HPV infection and PCa and found a statistically significant difference between the PCa patient group and the control group. One of the early studies was conducted by Anwar et al. (8) who identified 28 different HPV types in 68 PCa samples and reported that all BPH control samples were HPV DNA negative. Sert et al. (9) used a quantitative competitive PCR method to detect HPV 16/E6 and reported a significantly higher number of HPV 16/E6 DNA copies in the PCa samples (10 out of 47 samples, 21%) compared with BPH tissues (1 of 37 samples, 3%).

Leiros et al. (10) reported that 17 (41.5%) of 41 PCa samples (transrectal biopsy samples) were HPV DNA positive whereas none of the BPH samples contained HPV DNA (p<0.0001). Martinez-Fierro et al. (13) found the prevalence of HPV DNA to be 20% in PCa samples and 5.3% in the control samples, and suggested a significant relationship between the risk of PCa and the presence of HPV sequences.

Cuzick and Strickler suggested that early studies reported a higher positivity compared with the results of recent studies which found more negativity, and this might be due to HPV contamination with nearby tissues during sampling since HPV DNA was detected in the urethral and anal tissues. Based on this information, some researchers suggested using tissues obtained from radical surgery and microdissection of the neoplastic sample (28, 29).

In the current study, to prevent the HPV contamination of transurethral approach, all samples were obtained using open radical prostatectomy (for the PCa samples) and transvesical open prostatectomy (for the BPH samples). Furthermore, all the tumor samples were reviewed for a second time by two expert pathologists and only the sections that contained tumor cells were included in the analysis of the presence of HPV DNA.

The remaining 36 studies in the literature, however, did not report any difference between the PCa patients and the control cases in terms of the presence of HPV DNA. In the current study, only 1 of 60 PCa samples was found to be HPV DNA positive whereas all 36 BPH samples were HPV negative. Using the pyrosequencing analysis, the type of HPV positive sample was identified as HPV-57, which is considered to be low-risk and associated with skin lesions. A retrospective assessment of the patient with HPV-57 showed that the patient had genital wart. It was considered that the PCa sample was contaminated during the open radical prostatectomy. Despite the difficulty of presenting conclusive evidence for negative results, our DNA samples were of sufficient quality to amplify the human control gene (β-globin), and the internal amplification control showed that the extraction protocol did not inhibit the PCR.

The data obtained in the current study is in agreement with the results of two studies; one by Bergh and the other by Sfanos. Bergh analyzed 352 samples (171 PCa and 181 BPH) and Sfanos investigated 200 PCa samples. Neither detected HPV-DNA in prostate tissue samples (11, 12).

Storey et al. (1998) showed that the p53 homozygotes pose a high risk for the patients in terms of developing HPV-associated cervical cancer (30). Since then, a considerable number of studies have been conducted to explore the association between the p53 codon 72 polymorphism and various cancer types such as breast, pancreas, colorectal, lung and bladder cancer; however, these studies reported contradictory results (17).

In recent years, researchers have suggested that the p53 codon 72 polymorphism has a significant role in the development of tumors and the progression of PCa; however, contradictory results have been reported (18-22). Wu et al. (21) found that the Pro genotype is 2.6 times more frequent than the Arg variant in patients with PCa and the difference was statistically significant. In another study by Henner et al. (18), p53 Pro homozygosity in men reduced the risk of developing PCa and therefore Pro allele can have a protective effect. Doosti and Dekhordi (22) found a significant difference between PCa patients and the control group in terms of the Arg/Arg genotype and the frequency of Arg allele, and suggested that the Arg/Arg genotype can be a risk factor for the development of PCa in Southest Iran.

On the other hand, four studies did not find any association between the p53 codon 72 polymorphism and PCa. Huang et al. (20) suggested that there is no correlation between PCa and the p53 codon 72 polymorphism and put forward the hypothesis that p21 codon 31 polymorphism is associated with both the development of PCa and BPH. Leiros et al. (10) concluded that there is no correlation between p53 codon 72 polymorphism and HPV positive and negative PCa and hyperplasia. Salehi and Hadavi (31) reported no significant difference between the tumor and control groups and concluded that neither the p53 codon 72 polymorphism nor HPV infection results in susceptibility to PCa in an Iranian population.

Michopoulou et al. (2014) who conducted a study with 50 samples obtained from a Greek population, found HPV positivity in 8 PCa samples (16%) and 1 control sample (3.3%) using the real-time PCR method. Furthermore, the authors explored p53 codon 72 polymorphism in the same patients and found the distribution of three genotypes namely Arg/Arg, Arg/Pro, and Pro/Pro to be 69.6, 21.7, and 8.7%, respectively in the PCa group, and 75.0, 17.86, and 7.14%, respectively in the healthy control group. The authors did not report a statistically significant difference between HPV presence and factors such as age, stage, p53 codon 72 polymorphism and PCa (32).

Due to these contradictory results and the lack of reliable data on the genotype distribution of the p53 codon 72 polymorphism in a Turkish population, we conducted the current research with PCa patients and a control group. Comparing the genotype frequency of the two groups, it can be postulated that in the Turkish population, the Arg/Pro genotype and Pro alleles are more frequently seen in patients with prostate cancer.

The apoptosis-stimulating proteins (ASPP) of the p53 family regulate the function of apoptosis of the p53 codon 72Arg / Pro polymorphism. The apoptosis function of the p53 codon 72Pro variant is selectively inhibited by apoptosis-stimulating protein inhibitors (iASPP). The strong capacity of the p53 codon 72Arg variant to induce apoptosis results from its ability to escape from iASPP inhibition and its even greater abilitiy to localize to the mitochondria. Apoptosis is less frequently seen in people with the p53 codon 72Pro/Pro genotype compared with those with the Arg/Arg genotype and therefore Pro allele is more susceptible to the development of cancer (33). This is also supported by the results of the current study.

As in all research studies, this study also has certain limitations. Since the tissue samples had been obtained from patients with localized prostate cancer treated with radical prostatectomy, the number of samples was relatively smaller and thus the statistical power and potential bias of the study were limited. We did not have access to these samples because surgical treatment is not among the treatment options for advanced stage prostate cancer and metastatic cases. Furthermore, prostate biopsy is used in the follow-up of patients with high grade PIN; therefore, the tissues of these patients were not available. In addition to that this was a retrospective study, in which we did not have access to the blood samples of the patients, we could not perform an HPV serology. The last limitation of the study was that the control group consisted of samples with BPH; however, a control group with normal prostate tissue may have been more appropriate to show the association between prostate carcinogenesis and HPV infection.

## CONCLUSIONS

This is the first study that investigated the etiological role of HPV and the p53 codon 72 polymorphism in the development of PCa in a Turkish population. The results of the present study do not support the hyphothesis that prostate cancer is associated with HPV infection but indicate that Proline allele can be a risk factor for the development of PCa in the Turkish population. Further studies with larger series are needed to investigate the potential role of HPV in prostate carcinogenesis.

## ARTICLE INFO

Int Braz J Urol. 2017; 43: 36-47
